# Multifocal pyoderma gangrenosum mimicking disseminated cutaneous leishmaniasis ‒ a diagnostic challenge^☆^

**DOI:** 10.1016/j.abd.2024.04.013

**Published:** 2025-01-22

**Authors:** Ana Flávia Mundim Ramos, Cristiane Botelho Miranda Cárcano, Davi Carvalho Brito, Daniele Moraes Losada, Cristina Alessi da Rocha, Bruno Augusto Alvares

**Affiliations:** a Department of Dermatology, Santa Casa de Misericórdia de Barretos, Barretos, SP, Brazil; b Department of Dermatology, Hospital de Câncer de Barretos, Barretos, SP, Brazil; c Department of Pathology, Hospital de Câncer de Barretos, Barretos, SP, Brazil; d Department of Dermatology, Faculdade de Ciências da Saúde de Barretos Dr. Paulo Prata, Barretos, SP, Brazil

Dear Editor,

Pyoderma gangrenosum (PG) is a neutrophilic inflammatory dermatosis caused by the dysregulation of innate and adaptive immune components in genetically predisposed individuals.^1^ In Latin America, recognizing PG becomes more challenging, since skin and soft tissue infections often mimic this dermatosis.^2^ This report describes a case of PG with a previous histopathological diagnosis suggestive of cutaneous leishmaniasis.

A 49-year-old male patient from Rio de Janeiro presented with a one-year history of skin ulcers. The lesions started as pustules located on the left shoulder and cervical region, which rapidly developed into painful ulcers with a purulent exudate, affecting multiple areas of the skin (Fig. 1, Fig. 2). Asthenia, polyarthralgia, and edema of the upper limbs were reported. He had a history of an anal fistula, which preceded the skin manifestations, in addition to chronic diarrhea. Histopathology performed at another institution suggested a diagnosis of leishmaniasis, so two cycles of treatment with meglumine antimoniate were carried out, but there was lesion recurrence. A new therapeutic attempt with miltefosine resulted in worsening of the clinical picture.

**Fig. 1:**
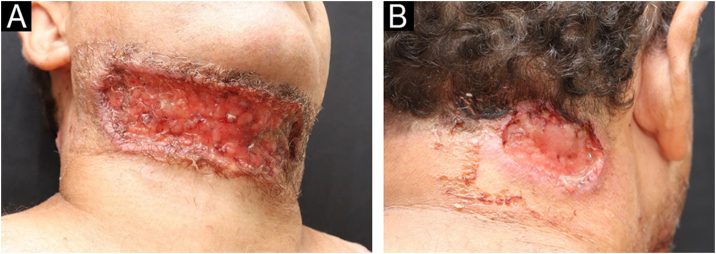
Ulcers with a granular bottom and undermined erythematous-violaceous edges in the anterior cervical region (A) and right side of the posterior cervical region (B).

**Fig. 2:**
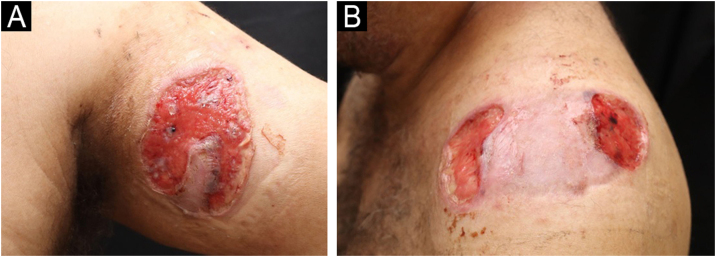
Lesions in the anterior region of the left arm (A) and left shoulder (B).

A new anatomopathological examination was performed, which showed a predominantly neutrophilic, perivascular, and periadnexal nonspecific inflammatory infiltrate. Serch for fungi (Grocott) and micobacteria (Fite-Faraco and Ziehl-Neelsen) were negative and the immunohistochemical technique for Leishmania spp. was negative. Serology and immunoelectrophoresis for fungi and autoantibodies were negative.

Considering the histopathology suggestive of a neutrophilic dermatosis, the clinical picture of painful ulcers, complains suggesting an inflammatory bowel disease (IBD), and complementary tests without evidence of infection, the diagnosis of PG was established.

The patient responded well to the combination of prednisone 1 mg/kg/day and doxycycline 100 mg/day but relapsed when there was an attempt to wean off the corticosteroid. After confirmation of the diagnosis of Crohn's disease, infliximab 5 mg/kg every eight weeks was started, and after six months, the patient showed complete remission of the skin and anal lesions (Fig. 3).

**Fig. 3:**
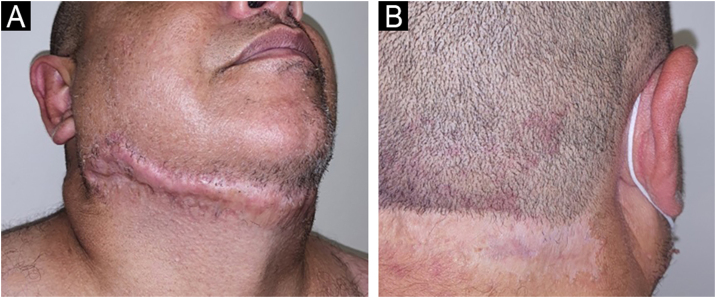
Complete healing of anterior (A) and right posterior (B) cervical lesions after treatment with infliximab.

Ulcerative PG is the most common clinical presentation associated with IBD in Latin America.^2^ The clinical manifestations, histological findings, and laboratory tests are not very specific, and in the past, PG was considered a “diagnosis of exclusion.”^3^, ^4^ To help in the evaluation, Maverakis et. al. postulated major and minor criteria for the diagnosis of PG through a Delphi consensus. According to these criteria, one major criterion (biopsy of the ulcer edge demonstrating a neutrophilic infiltrate) and at least four of eight minor criteria (exclusion of infection; pathergy; history of IBD or inflammatory arthritis; history of papule, pustule, or vesicle with rapid progression to ulceration; peripheral erythema, ulceration with undermined, and tenderness at the ulceration site; multiple ulcerations, at least one on the anterior leg; cribriform or “crumpled paper” scar at the sites of the healed ulcer; and decrease in ulcer size within one month after starting immunosuppressive medication) are required for the diagnosis of PG.^4^

Ruling out infections is crucial for the diagnosis of PG, since immunosuppressive therapies may be contraindicated in these situations. The diagnostic challenge in the present case is related to patient epidemiology and the previous histopathology reporting the presence of structures that could correspond to amastigotes or degenerated neutrophils.

In the cutaneous form of leishmaniasis, micrometric structures corresponding to amastigotes are found inside macrophages. Ulceration is frequent in acute lesions, and the dermis typically contains an abundant inflammatory infiltrate composed by histiocytes, lymphocytes, and plasma cells, but with few in neutrophils and eosinophils. There may be foci of dermal necrosis.^5^

PG exhibits histomorphological variation depending on the location and chronology of the lesion. In general, the lesions manifest as nonspecific ulcerative processes, with the formation of neutrophilic abscesses. Secondary acute necrotizing vasculitis may be observed and leukocytoclasia may occur. Giant cells are observed in patients with PG and Crohn's disease (Fig. 4).^5^

**Fig. 4:**
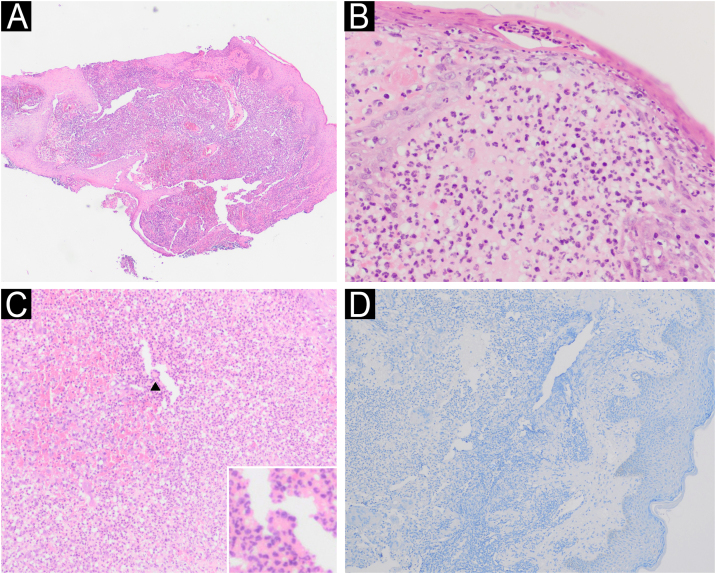
(A) Histopathology of ulcer edge showing a dense neutrophilic inflammatory infiltrate (Hematoxylin & eosin, ×4). (B) Subcorneal pustule and neutrophilic abscess in the papillary dermis (Hematoxylin & eosin, ×40). (C) Neutrophilic inflammatory infiltrate (Hematoxylin & eosin, ×20); multinucleated giant cell in detail (arrow). (D) Negative immunohistochemistry for Leishmania spp.

Despite the initial diagnostic uncertainty, upon reviewing the case and considering the diagnostic criteria of the Delphi consensus,^4^ the patient in the present case met the necessary criteria for the diagnosis of PG: histological findings (major criterion), absence of infection, active IBD, multiple and painful ulcers with undermined edges and a favorable clinical response to immunosuppression (minor criteria).

The present case highlights the importance of clinical-pathological correlation and the application of validated criteria for the assertive and early diagnosis of patients with PG.

## Financial support

None declared.

## Authors' contributions

Ana Flávia Mundim Ramos: Drafting and editing of the manuscript or critical review of important intellectual content; intellectual participation in the propaedeutic and/or therapeutic conduct of the studied cases; critical review of the literature.

Cristiane Botelho Miranda Cárcano: Intellectual participation in the propaedeutic and/or therapeutic conduct of the studied cases; approval of the final version of the manuscript.

Davi Carvalho Brito: Intellectual participation in the propaedeutic and/or therapeutic conduct of the studied cases; critical review of the literature.

Daniele Moraes Losada: Intellectual participation in the propaedeutic and/or therapeutic conduct of the studied cases; approval of the final version of the manuscript.

Cristina Alessi: Effective participation in research orientation; approval of the final version of the manuscript.

Bruno Augusto Alvares: Intellectual participation in the propaedeutic and/or therapeutic conduct of the studied cases; effective participation in research orientation; approval of the final version of the manuscript.

## Conflicts of interest

None declared.
